# Correction: Baradad-Jurjo et al. Measurement of Melanocytic Choroidal Lesions: Ultrasound Versus Ultrawide-Field Fundus Imaging System. *Cancers* 2025, *17*, 642

**DOI:** 10.3390/cancers17152463

**Published:** 2025-07-25

**Authors:** Maria C. Baradad-Jurjo, Daniel Lorenzo, Estel·la Rojas-Pineda, Laura Vigués-Jorba, Rahul Morwani, Lluís Arias, Pere Garcia-Bru, Estefania Cobos, Juan Francisco Santamaria, Carmen Antia Rodríguez-Fernández, Josep M. Caminal

**Affiliations:** 1 Ophthalmology Department, Bellvitge University Hospital, Feixa Llarga Street, s/n, 08907 Hospitalet de Llobregat, Spain; dlorenzo@bellvitgehospital.cat (D.L.); rpstela@gmail.com (E.R.-P.); rahulmorwani89@gmail.com (R.M.); peregarciabru@gmail.com (P.G.-B.); ecobosmartin@gmail.com (E.C.); juanfra_s@hotmail.com (J.F.S.); carmenantia@gmail.com (C.A.R.-F.); jmcaminal@gmail.com (J.M.C.); 2 Facultat de Medicina, Campus Bellvitge, Universitat de Barcelona (UB), Feixa Llarga Street, s/n, 08907 Hospitalet de Llobregat, Spain

## Additional Affiliation

In the published publication [1], there was an error regarding the affiliation for Maria C. Baradad-Jurjo. In addition to affiliation number 1, the updated affiliations should include the following: Facultat de Medicina, Campus Bellvitge, Universitat de Barcelona (UB), Feixa Llarga Street, s/n, 08907 Hospitalet de Llobregat, Spain.

The authors state that the scientific conclusions are unaffected. This correction was approved by the Academic Editor. The original publication has also been updated.
